# Inflammatory Myofibroblastic Tumor of the Tongue: A Rare Case in a Pediatric Patient

**DOI:** 10.7759/cureus.92373

**Published:** 2025-09-15

**Authors:** Caterina Unia, Claire De Rosnay, Vianney Ribeiro, Pierre Sohier, François Le Pelletier, Ihsène Taihi

**Affiliations:** 1 Oral Surgery, Université Paris Cité, Paris, FRA; 2 Odontology, Université Paris Cité, Paris, FRA; 3 Oral Surgery, Hôpital Rothschild, Paris, FRA; 4 Pathology, Hôpital Cochin, Paris, FRA; 5 Pathology, Pitié-Salpêtrière Hospital, Paris, FRA; 6 Orofacial Pathology, Imaging, and Biotherapy, Université Paris Cité, Paris, FRA

**Keywords:** connective and soft tissue, fluorescence in situ hybridization (fish), neoplasms, targeted drug therapy, tongue neoplasms

## Abstract

Inflammatory myofibroblastic tumors (IMTs) are rare neoplasms with intermediate biological potential, most often found in the abdominal cavity of children and young adults. Oral IMTs are extremely rare, presenting diagnostic challenges due to non-specific clinical features and a wide range of differential diagnoses. A 13-year-old female presented with an asymptomatic nodule on the dorsal tongue. Surgical excision was performed. Histopathological analysis, immunohistochemistry, and in situ hybridization identified spindle-shaped myofibroblastic cells, an inflammatory infiltrate, low proliferative activity (Ki-67), and an *ALK* gene rearrangement. These findings confirmed the diagnosis of oral IMT with a favorable prognosis. The surgical excision was complete. This case highlights the diagnostic complexity of oral IMTs and underscores the importance of histopathological and genetic analysis in establishing prognosis and identifying potential therapeutic targets.

## Introduction

Inflammatory myofibroblastic tumors (IMTs) are rare neoplasms classified by the World Health Organization (WHO) within myofibromatous tumors in the International Classification of Diseases for Oncology, third edition (ICD-O-3), and belong to chapter 881-883 of myofibromatous tumors. They are considered intermediate neoplasms because of their possible recurrence and metastatic potential [1]. Histologically, they are composed of myofibroblastic and fibroblastic spindle cells accompanied by inflammatory cells, such as lymphocytes, plasma cells, and eosinophils [2]. The classification of IMT as a true neoplasia or “pseudotumor” has been a predominant debate in the past, due to its nonspecific appearance. Due to their rarity and uncertain prognosis, clinical guidelines for IMTs remain inconsistent.

The precise prevalence of IMT is challenging to determine, owing to its nonspecific clinical presentation and historical misclassification. However, it is estimated to be fewer than 1% of all soft tissue tumors. IMTs mostly occur in the soft tissues of the abdominal cavity (75% of cases), specifically in the mesentery, greater omentum, and retroperitoneal space. They can also be found in the lungs, neck, and bladder [3]. They usually affect children and young adults, but can be found at every age [4]. Their recurrence depends on the location, varying from 2% for pulmonary lesions up to 25% for extrapulmonary lesions [3]. Their etiopathogenesis is still unclear, but various gene rearrangements have been found. The presence of a gene rearrangement, particularly the anaplastic lymphoma kinase (*ALK*) gene, is a key element, confirming the diagnosis and differentiating IMT from other pathological entities. The identification of these gene rearrangements can greatly contribute to a better understanding of this disease and finding a targeted therapy, potentially leading to lower recurrence rates. These findings have also been instrumental in refining the diagnostic criteria and prognostic stratification.

In this article, we report a rare case of lingual IMT in a pediatric patient, highlighting the diagnostic challenges, treatment considerations, and the importance of genetic analysis in guiding treatment. This work aims to contribute to the growing understanding of this rare entity and provide clinicians with practical insights into its diagnosis and management.

## Case presentation

A 13-year-old female presented to the oral dermatology department in July 2020, accompanied by her mother, with a complaint of a swelling on the dorsal face of her tongue. The patient was in good overall health, with no history of chronic treatment and no systemic symptoms such as fever, fatigue, appetite loss, or weight loss. The lesion, which appeared one month prior, had not shown any reduction in size. The patient did not report pain, paresthesia, burning, or taste disturbances, although slight tingling was noted when consuming acidic foods. The mother mentioned the patient had a habit of tongue biting, though it was unclear if this was a cause or a contributing factor to the swelling.

**Figure 1:**
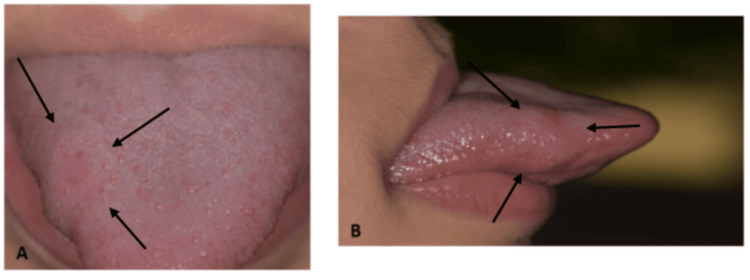
Intraoral view of the tongue of the patient: (A) upper side and (B) lateral side. The arrows indicate the lesion.

Clinical examination revealed no cervical or facial lymphadenopathy, no trismus, no fistula, and normal skin. Intraorally, a single, raised, well-circumscribed, erythematous nodule was observed on the dorsal and right lateral surface of the tongue, approximately 1 cm in diameter (Figures 1A, 1B).

The nodule was non-tender on palpation. Given the absence of systemic symptoms or significant findings, no additional imaging or biological tests were initially performed.

**Figure 2:**
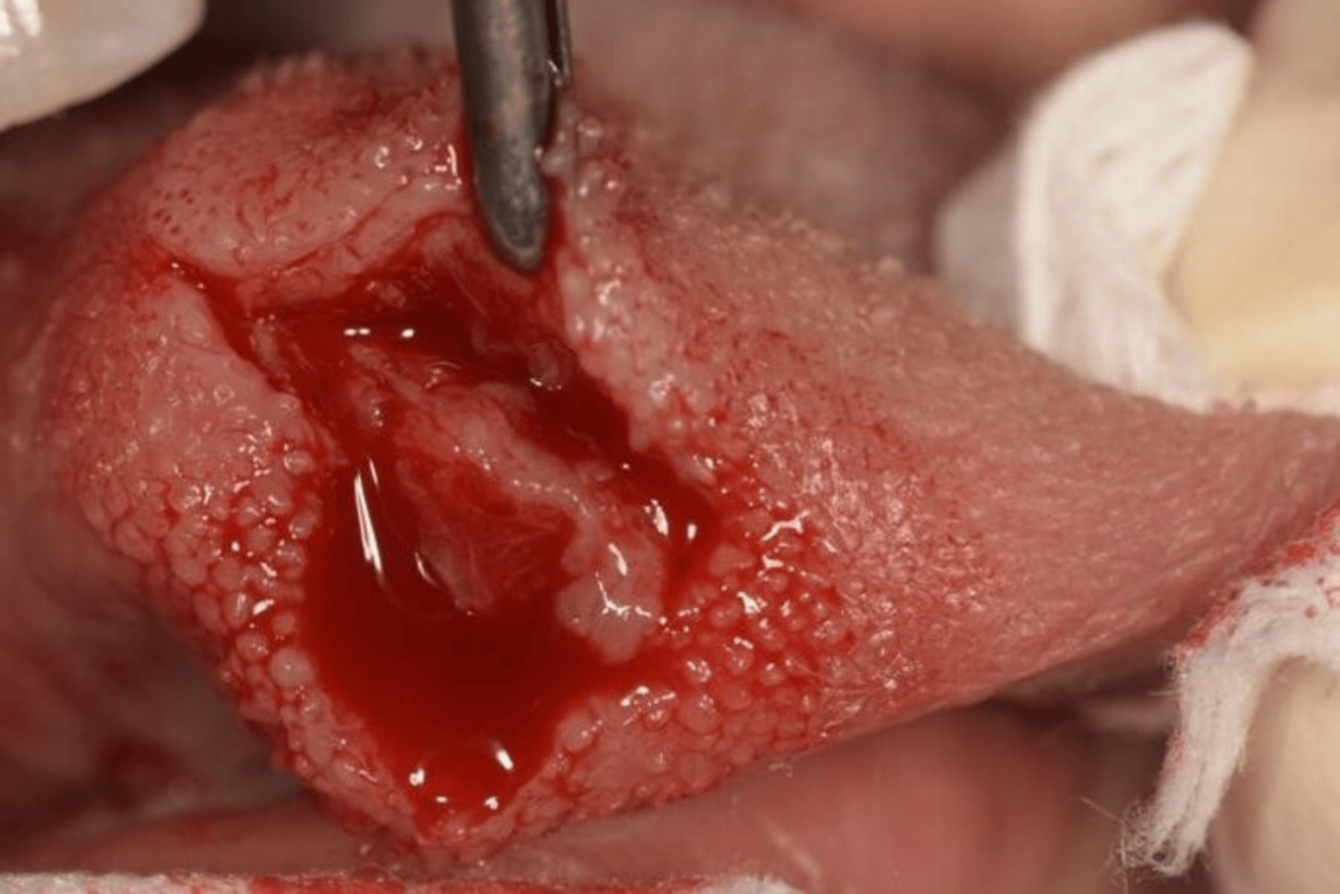
Clinical view of the inflammatory myofibroblastic tumor during surgical resection.

The main differential diagnoses included leiomyosarcoma, embryonal rhabdomyosarcoma, fibrosarcoma, myofibroblastic sarcoma, and liposarcoma. Other hypothetical diagnoses were also mentioned, including cysts or pseudocysts (e.g., mucocele) and benign mesenchymal tumors such as fibromas or lipomas. An en bloc excision of the lesion was performed under local anesthesia, and the sample was sent for pathological analysis. The tumor was noted to be adherent to surrounding tissue (Figure 2).

**Figure 3:**
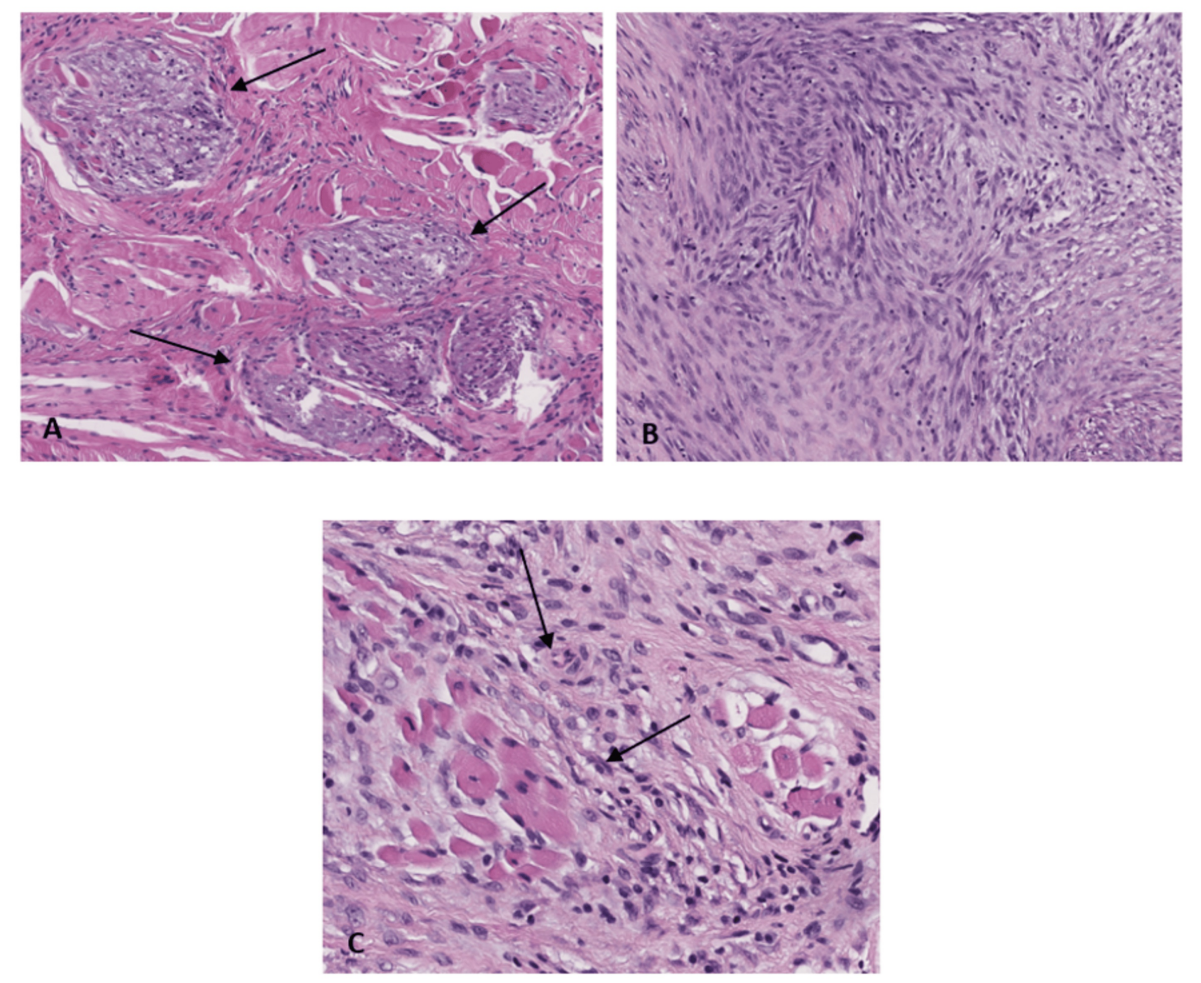
(A) Inflammatory myofibroblastic tumor (hematoxylin and eosin, ×120): poorly demarcated multinodular profileration. (B) Inflammatory myofibroblastic tumor (hematoxylin and eosin, ×120): spindle cells. (C) Inflammatory myofibroblastic tumor (hematoxylin and eosin, ×250): low inflammation and lymphocyte nuclei.

Histopathological examination confirmed clear surgical margins and complete excision of the lesion. Moreover, it revealed poorly demarcated cellular proliferation within the chorion, organized in nodular patterns with variable cellular density. Some areas showed an edematous stroma with sparse cells, while others were fibrous with slightly higher cellularity. The spindle-shaped cells displayed eosinophilic to amphophilic cytoplasm, fine chromatin, and small central nucleoli. Sparse lymphocytic infiltration and occasional multinucleated giant cells were noted, with the proliferation extending into lingual striated muscle fibers and evidence of atrophic muscle. No significant mitotic activity was present. Certain cells exhibited a pseudoganglionic appearance (Figure 3). While characteristic, these histological findings were not sufficient for a positive diagnosis, due to the possibility of an overlap with other lesions. An immunohistochemical analysis was then conducted.

**Figure 4:**
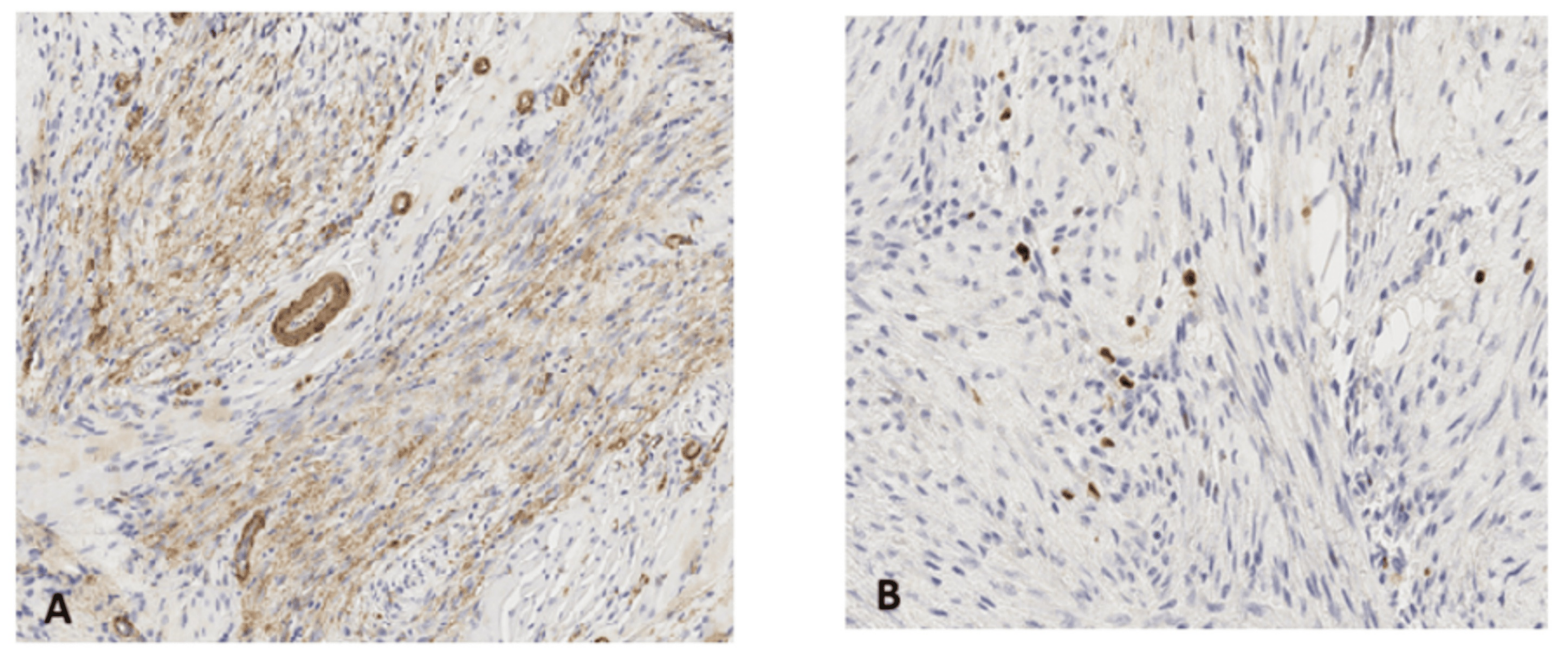
(A) Histological section (hematoxylin and eosin, ×120) showing positive marking for smooth muscle actin. (B) Ki-67 marking and low proliferation index at 5% (hematoxylin and eosin, ×210).

Immunohistochemical analysis indicated negative staining for AE1/AE3, EMA, PS100, desmin, CD34, and caldesmon, with positive staining for smooth muscle actin (SMA) (Figure 4A). ALK immunohistochemistry revealed diffuse cytoplasmic positivity. Ki-67, a marker of cellular proliferation, was weakly expressed (5%), supporting a non-malignant diagnosis (Figure 4B).

**Figure 5:**
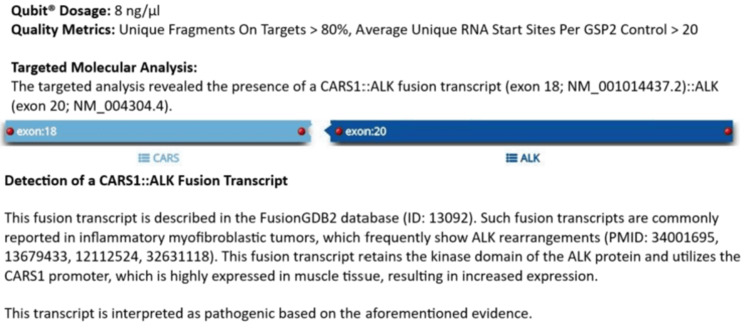
Next-generation sequencing showing ALK gene rearrangement with CARS1.

The next-generation sequencing confirmed an *ALK* gene rearrangement with the *CARS1* gene (Figure 5). This gene is located within the imprinted gene domain on chromosome 11p15.5, a critical region for its tumor-suppressor function. Alterations in this region have been implicated in various conditions, including microcephaly, Beckwith-Wiedemann syndrome, Wilms tumor, rhabdomyosarcoma, adrenocortical carcinoma, as well as lung, ovarian, and breast cancers. This fusion transcript between *ALK* and *CARS1* confers oncogenic potential to *ALK* and provides insights into the pathogenesis of the lesion, confirming its diagnosis as an oral IMT. *USP6* gene rearrangement was negative.

**Figure 6:**
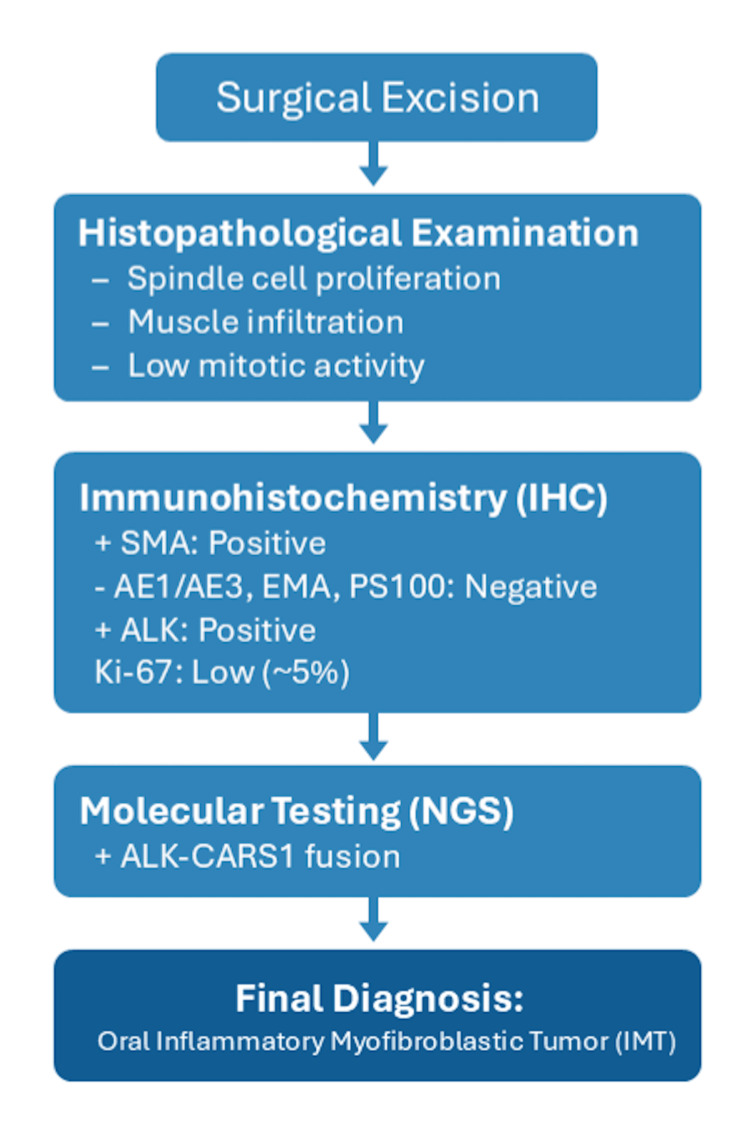
A summary of the diagnostic procedure.

Diagnosing IMT can be difficult because its nonspecific clinical and histological features can lead to other similar pathological entities. Each step of the diagnostic procedure helps narrow it down and confirm IMT: first clinical examination and imaging, then histopathological analysis, and, ultimately, immunohistological analysis and molecular testing (Figure 6). Molecular testing in IMT is relevant in both the diagnostic and treatment plans. It helps identify gene rearrangements such as *ALK*, thus confirming the diagnosis and distinguishing IMT from other lesions. In *ALK*-negative cases, molecular testing can reveal other gene rearrangements. Finding these rearrangements is key to targeted therapy, which could improve treatment and prognosis.

Recurrence of the lesion was observed for this patient after one year of follow-up. However, the parents did not come back for the second excisional surgery appointment. Her complicated family circumstances prevented long-term follow-up.

## Discussion

The clinical challenge in diagnosing IMTs in oral localization arises from their nonspecific presentation, necessitating the consideration of various differential diagnoses, particularly other mesenchymal lesions. Moreover, the terminology of IMTs has evolved over the past few years, with the WHO classifying them as neoplastic diseases with intermediate biological potential due to their risk of recurrence and metastasis. The recurrence rate varies according to anatomical site, with extrapulmonary lesions having a higher recurrence rate than pulmonary lesions [5]. Concerning the prognosis of oral IMT, it seems to be better than extraoral localizations. Indeed, the review conducted by Brooks et al. (2005) [6] showed that 15 cases of IMT located in the oral mucosa, in different localizations, including the jugal mucosa, parotid gland, submandibular region, alveolar mucosa, and tongue, have a good prognosis with no recurrence or metastasis.

In the literature, specific symptoms are described according to the region of involvement and can be reported for each region. For example, in extraoral localizations, pulmonary IMT may be associated with chest pain and dyspnea but may also remain asymptomatic. Abdominal IMT is likely to cause gastrointestinal obstruction. In 15-30% of cases, general clinical signs occur with fever, stunting, malaise, and/or weight loss.

Regarding oral IMTs, the clinical aspect described in the different publications often consists of a painless induration, with a pedunculated or polyploid, yellowish-white, firm, or gelatinous base, rarely measuring more than 3 cm in diameter. IMTs are often non-encapsulated, with polycyclic outlines, with the occasional presence of central necrosis. Hemorrhagic areas and calcifications are possible. An inflammatory syndrome with anemia, thrombocytosis, polyclonal hyperglobulinemia, and elevated erythrocyte sedimentation rate may be noted [7]. It has been reported that when the mass is excised, these signs disappear, and their reappearance denotes a recurrence. Imaging studies (MRI, CT, or ultrasound) reveal a solid lobulated mass that may be inhomogeneous. Calcifications are sometimes detectable.

In the WHO classification of soft tissue tumors, IMTs are characterized by a proliferation of spindle-shaped myofibroblastic cells associated with an inflammatory infiltrate composed of plasma cells, lymphocytes, and eosinophils [8]. Histologically, it is a proliferation of spindle cells with eosinophilic cytoplasm corresponding to myofibroblasts. The nuclei may be large, sometimes atypical, and have a prominent nucleolus. An inflammatory infiltrate composed mainly of plasma cells, lymphocytes, and eosinophilic polynuclears is found in typical cases. The stroma is sometimes loose, edematous, or myxoid, and may have a significant number of blood vessels [9]. Mitotic activity is variable.

Immunohistochemically, IMT commonly expresses vimentin (95-100% of cases), desmin (5-80% of cases), AML (48-100%), keratin (10-89%), and ALK (35-60% of cases) [5]. ALK is a receptor tyrosine kinase and a member of the insulin growth factor receptor superfamily. It is only expressed in neural tissue under normal conditions. Clonal abnormalities of ALK are typically associated with anaplastic large cell lymphoma (ALCL), which has a chromosomal translocation, t (2;5) (p23; q35), in approximately 40% of cases [10]. About 50% of IMTs also have ALK rearrangements, demonstrated by fluorescent in situ hybridization. Owing to this method, several ALK rearrangements have been identified, notably *TPM3* at 1p23, *TPM4* at 19p13, *ATIC* at 2q35, *CLTC* at 17q23, *CARS* at 11p1, RANBP2 at 2q13, and *SEC31L1* at 4q216-8 [11-13]. The rearrangements involving the ALK locus point to a neoplastic rather than inflammatory or reactive origin of IMT and their distinction from other inflammatory pseudotumors.

Only histological and immunohistochemical analysis can confirm the diagnosis of IMT and whether the excision performed is complete. If the excision is complete, a surveillance can be set up. If it is incomplete and a new operation is too mutilating, it is important to know the nature of the IMT (ALK positive, negative, with or without known genetic rearrangement) to prescribe the most appropriate complementary drug treatment. The presence of ALK rearrangement appears to be a favorable prognostic indicator, associated with fewer recurrences and distant metastases [1,5,14]. Furthermore, the prognosis improves when the IMT expresses this ALK rearrangement and is completely excised [15]. Therefore, the detection of an ALK fusion transcript is a valuable diagnostic and prognostic marker and enables the implementation of targeted therapeutic strategies.

Marie-Cardine et al. suggest that an extension workup should be performed if several sites are involved. This assessment includes a CT scan of the lungs and mediastinum, an abdominal ultrasound scan, and a brain MRI. Bone X-rays or bone scans may be performed if there are clinical signs of bone disease [16]. However, this work-up has not been mentioned in any of the studies dealing with oral and particularly lingual IMT. Surveillance should be clinical and radiological and should include all sites initially affected for a period of up to 10 years. More specific surveillance may be instituted when IMT is ALK negative or when excision is incomplete [6].

Complete excision seems to be the treatment of choice with good results [17,18]. In cases of recurrent IMT or when the benefit-to-risk ratio of excision is unfavorable, treatment with a tyrosine kinase inhibitor may be indicated, such as crizotinib (a first-generation tyrosine kinase inhibitor). It appears to be an effective treatment for *ALK*-positive IMT [19] but also for *ALK*-negative IMT with a rearrangement of the *ROS1* gene, probably because *ROS1* is structurally similar to ALK [19]. For other *ALK*-negative but *NTRK*-positive IMTs, larotrectinib can be used as an *NTRK* inhibitor. When a *PDGFR* gene rearrangement is found, the drug treatments used in the cases described in the literature are sorafenib, sunitinib, regorafenib, and axitinib. Finally, when the rearrangement concerns the *RET* gene, treatment with a multi-targeted tyrosine kinase inhibitor with anti-*RET* activity (sunitinib, sorafenib, or vandetanib) may be used [19].

## Conclusions

This case report emphasizes the challenges involved in diagnosing and managing lingual IMTs due to their rarity and nonspecific clinical presentation. To date, there are no specific recommendations or guidelines regarding the management of these lesions. However, available evidence suggests that their prognosis is generally more favorable compared to extraoral localizations such as pulmonary or urological forms. The treatment of choice consists of the most complete surgical removal possible. If it is incomplete and a new operation is too mutilating, understanding the biological nature of the IMT, specifically its ALK status, is crucial to guide appropriate adjunctive therapies, including targeted drug treatments. Close follow-up must be set up, although oral IMT appears to have a low recurrence rate. This case shows the importance of thorough histopathological and genetic analyses to differentiate IMTs from other neoplastic or inflammatory lesions, which ensures accurate diagnosis and appropriate treatment. Given the limited data on oral IMTs, further research is needed to refine their clinical behavior and prognostic markers and develop evidence-based management strategies. Long-term follow-up of affected patients will be invaluable in enhancing our understanding of these rare lesions, optimizing treatment, and improving outcomes.
